# Effect of Canal Anastomosis on Periapical Fluid Pressure Build-up during Needle Irrigation in Single Roots with Double Canals using a Polycarbonate Model

**DOI:** 10.1038/s41598-017-01697-1

**Published:** 2017-05-08

**Authors:** Qi Huang, Jonathan B. Barnes, G. John Schoeffel, Bing Fan, Candice Tay, Brian E. Bergeron, Lisiane F. Susin, Jun-qi Ling, Li-na Niu, Franklin R. Tay

**Affiliations:** 1 Department of Operative Dentistry and Endodontics, Guanghua School of Stomatology, Sun Yat-sen University, Guangzhou, PR China; 2 Department of Endodontics, The Dental College of Georgia, Augusta University, Georgia, USA; 3 Retired, Dana Point, USA; 4 Department of Endodontics, School and Hospital of Stomatology, Wuhan University, Wuhan, China; 5 University of Toronto, Ontario, Canada; 6 State Key Laboratory of Military Stomatology & National Clinical Research Center for Oral Diseases & Shaanxi Key Laboratory of Oral Diseases, Department of Prosthodontics, School of Stomatology, The Fourth Military Medical University, Xi’an, PR China

## Abstract

Sodium hypochlorite is an effective irrigant for chemical debridement of root canals. However, increasing the intracanal pressure during irrigant delivery may result in irrigant extrusion into the bone and soft tissues surrounding the tooth. Because clinicians often encounter teeth with intracanal communications, the objective of the present study was to examine the effects of canal anastomosis on the generation of periapical fluid pressure at different fluid flow rates and insertion depths. Two similar polycarbonate models were used to simulate a single root with double canals, one containing, and the other without communicating channels between the canals. For both models, periapical pressure increased with increasing irrigant flow rates and insertion depths of a 30-gauge side-venting needle. In the presence of communicating channels, the magnitude of pressure build-up decreased by almost 90% irrespective of the fluid flow rate or needle insertion depth. Pressure reduction in anastomoses-containing roots provides an explanation why pressure generation in single roots is considerably higher. Nevertheless, it is still possible in teeth with canal anastomoses for pressure exceeding the intraosseous pressure to be generated when the fluid flow rate is sufficiently high and when the needle tip is close to the apical terminus.

## Introduction

Success in root canal treatment is dependent upon the clinicians’ ability to eradicate the source of intraradicular infection. Chemical debridement efficacy is related to the ability of the irrigant to infiltrate the entire canal space^1^. Irrigants are often introduced into root canals by positive pressure via a syringe attached to a hypodermic needle^2^. Sodium hypochlorite (NaOCl) is an effective irrigant for chemical debridement of root canals due to its ability to dissolve the organic components of necrotic and vital tissues, lubricate files during instrumentation, clear dentinal debris, and inactivate/dissolve bacterial biofilms^3^. The efficacy of NaOCl delivery is a function of the needle design, depth of irrigation needle insertion, and the flow rate of irrigant expressed from the needle^1, 2, 4^. The most difficult segment of a canal to be thoroughly irrigated is the apical third of the canal space. Even before endodontics became a dental specialty, anatomical studies have demonstrated that the greatest degree of complexity occurs in the apical third of the canal space^5, 6^.

There is a subtle balance between efficacy and safety for needle-assisted irrigation of the apical canal space. It is important to implement fluid exchange at the canal terminus by bringing the needle tip close to the working length of an instrumented canal, and by delivering the irrigant with a flow rate that is high enough to ensure bacteria and debris removal. However, the fluid flow rate of an irrigant, that of NaOCl in particular, must be controlled so that the irrigant flow is confined to, and not beyond the root canal system. There is a progressive increase in positive apical fluid pressure as irrigant flow rate increases inside the canal space^7–11^, to the point where the pressure is high enough to cause the irrigant to be extruded into the periapical tissues. Zhu *et al*. proposed a mechanism of NaOCl accident by intravenous infusion of the irrigant beyond the root apex into the sinusoids of the medullary bone^12^. This mechanism, analogous to the infusion of drugs via the bone marrow spaces^13^, occurs when the apical fluid pressure in a root canal exceeds 30 mm Hg (4 kPa; approximating intraosseous blood pressure). When there is a patent apical foramen, NaOCl may extrude beyond the confines of the tooth and in the presence of atypical venous drainage of a tooth to the facial venous system, a full-blown NaOCl accident may occur with rapid onset of facial oedema, pain and ecchymosis. Due to the difference between intraosseous blood pressure and the central venous pressure (1–7 mm Hg, or 0.13–0.93 kPa), the NaOCl that enters bone sinusoids rapidly drains to the superficial facial venous vasculature where ecchymosis is manifested.

To address both fluid exchange efficacy and safety concerns, closed-end side-venting type needles have been recommended for canal debridement, by inserting the hypodermal needle in a non-binding manner to 1 mm short of the working length^2^. Clinicians may be under the pretence that NaOCl accidents will not occur when a closed-end side-vented needle is not bound or locked in the canal. However, Khan *et al*. evaluated the fluid pressures generated in a single-canal resin model with various non-binding irrigation needles at various irrigant flow rates^14^. The authors opined that all positive pressure delivery systems tested were capable of generating enough apical pressure to exceed human intraosseous blood pressure (4 kPa) when the fluid flow rates were higher than 3–4 mL/min. In the single-canal polycarbonate block model used by those authors, the positive apical pressures generated by the use of a 30-gauge closed-end side-venting needle at 1 mm from the working length were much higher than those reported by Park *et al*. using the same needle type in a natural mandibular tooth root with two instrumented mesial canals^11^. In the work by Park *et al*., the highest positive periapical pressure obtained with insertion of a 30-gauge closed-end side vented needle to 1 mm from the working length was below 4 kPa even when high flow rates of 8 mL/min were used. Moreover, the positive periapical pressure generated with a fluid flow rate of 8 mL/min dropped to below 2 kPa when the needle was placed at 3 mm above the working length.

Both the models employed by Khan *et al*. and Park *et al*. contained canals that were prepared in non-compliant materials. That is, the materials (polycarbonate or root dentin) could not expand to accommodate the increase in fluid volume within the canal space. Although it was not readily apparent what accounted for the discrepancy in the magnitude of positive apical fluid pressure generated from those two studies, it is speculated that the two mesial canals prepared in the natural tooth utilised by Park *et al*. could have been connected by canal anastomosis. This could have resulted in the displacement of fluid from one canal to the other, thereby reducing the apical fluid pressure. Because clinicians often encounter teeth that contain single roots with multiple canals, which may contain an isthmus or other types of intracanal communication, the objective of the present study was to examine the effects of canal anastomosis on the generation of periapical fluid pressure at different fluid flow rates and needle insertion depths, using a polycarbonate model to simulate a single root with double canals. The null hypothesis tested was that canal anastomosis has no effect on the magnitude of periapical pressure build-up when a root canal irrigant is delivered at different fluid flow rates and needle insertion depths.

## Results and Discussion

**Figure 1:**
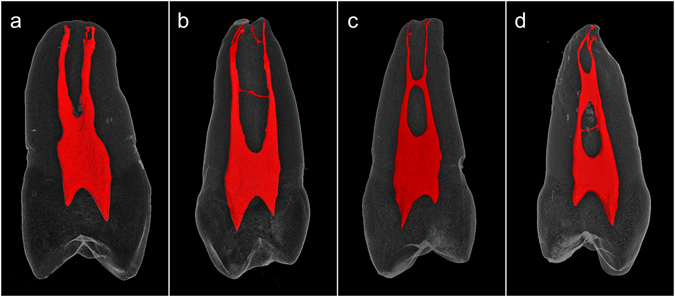
Representative micro-computed tomography reconstructions of single-rooted maxillary premolars containing double root canals. (**a**) Two separate canals. (**b**) Two separate canals connected by a lateral anastomosis in the mid-root. (**c**) Two separate canals connected by a larger anastomosis in the mid-root. (**d**) Two separate canals connected by a sheet-like isthmus in the middle-third and a narrow anastomosis in the coronal-third of the root.

Root canal anatomy in human teeth is highly complex, as demonstrated by previous dye and micro-computed tomography studies^15, 16^. To facilitate the development of clinically-relevant models that best approximate adjacent root canals with and without anastomoses, maxillary premolars with single roots were scanned with micro-computed tomography followed by 3-D reconstructions. One hundred and fifty-three out of the 200 randomly selected premolars were single-rooted premolars. Double canals could be identified in 84 of the single-rooted premolars, and 40.48% of those contained lateral anastomoses. Specifically, 17 of the 34 single-rooted double-canal first premolars (50%) and 17 of the 50 single-rooted double-canal second premolars (34%) had anastomosing connections in the form of discrete lateral channels and/or sheet-like isthmi. Representative examples are shown in Fig. 1. Videos of the 3-D rendered examples shown in Fig. 1 may be found as Supplementary Videos 1–4. Apart from differences in incidences, the information collected from human premolars with respect to how adjacent canals communicated within a single root should be applicable to mandibular incisors, mandibular premolars, as well as maxillary and mandibular molars that contain single roots with double canals^17–25^.

**Figure 2:**
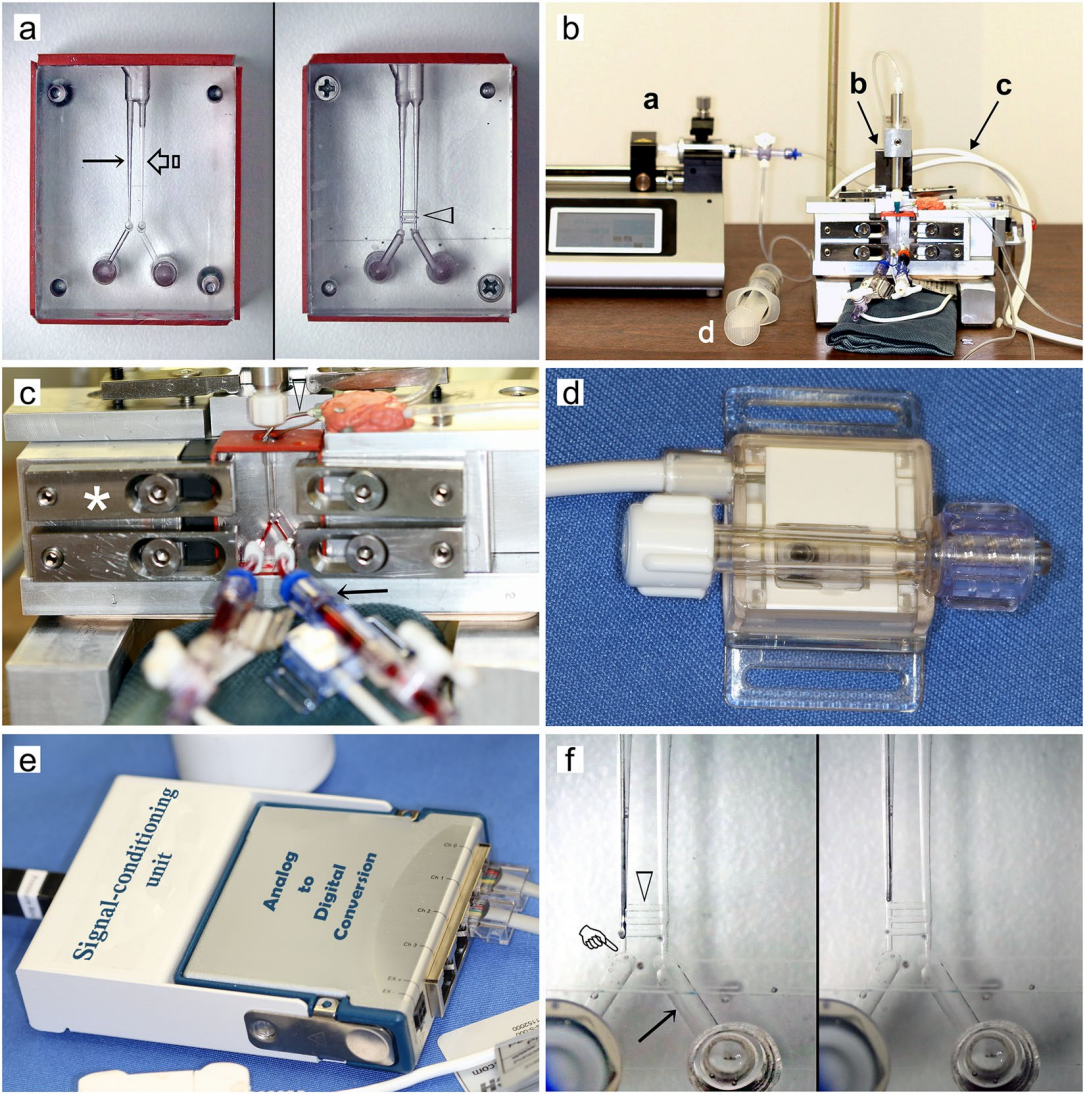
Experimental setup. (**a**) Two models simulating double canals in a single root. *Left*: The “separate canal model” with two unconnected parallel canals, one for pressure measurement with the irrigation needle inserted (arrow) and the other without needle insertion (open arrow). *Right*: The “canal anastomosis model” with a similar set of simulated parallel canals connected by anastomosing lateral channels (open arrowhead). The two holes at the bottom of each block are Luer ports for connecting the apical termination of each canal to a pressure sensor. (**b**) Overview of the experimental setup. b-a: precision syringe pump; b-b: Positioning device with inserted metal gauge blocks for precise control of the irrigation needle position within the canal space; b-c: Data acquisition cables linking the pressure sensors (Fig. 2d) to the signal-conditioning unit (Fig. 2e). b-d: 50 mL syringe connected to a two-way valve to refill the delivery syringe in the syringe pump. (**c**) The test unit with set-up clamps (asterisk) compressing against the polycarbonate split-block to achieve a fluid-tight seal. Solid arrowhead: aspiration needle for evacuating excess irrigant; Arrow: PVC tubing connecting a pressure sensor to the Luer port in front of the split-block (Fig. 2a) via a standard Luer connector. Red dye was added to the liquid within the tubing to demonstrate the path from the pressure sensor to the faux apical termination. (**d**) Pressure sensor that records the detected pressure in millivolts. (**e**) Signal-conditioning unit that converts millivolt analogue signals into digital signals. (**f**) Side-venting needles inserted into the anastomosing canal model. *Left*: Needle inserted to 1 mm above the apical termination of one canal (pointer). Open arrowhead: lateral channels connecting the two adjacent canals. Arrow: extension connecting the canal to the Luer port. *Right*: Needle inserted to 3 mm above the apical termination.

**Figure 3:**
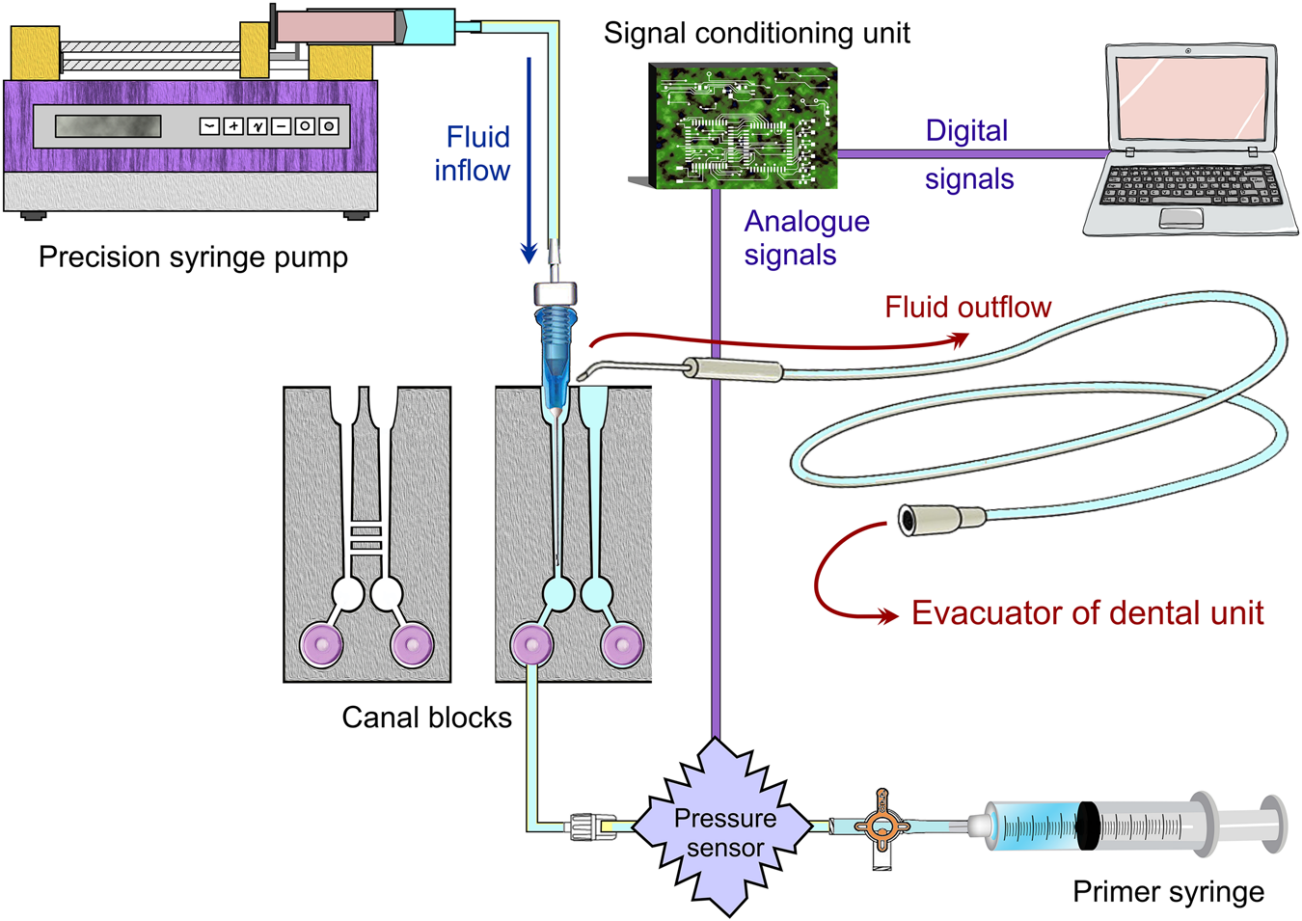
Schematic of fluid inflow-outflow and pressure signal recording of the experimental setup (http://www.corel.com/en/clipart-and-photos/). “Copyright (**c**) 2017 Franklin Tay and its licensors. All rights reserved.”

Based on the information derived from the 3-D rendered premolars, two models were created from polycarbonate blocks to simulate single roots with unconnected or connected double canals (Fig. 2a). The separate canal model was used to simulate highly-calcified canals that do not contain additional fins or cu-de-sacs. In this model, a second parallel, unconnected canal was used as the control canal. In the canal anastomosis mode, the two parallel canals were connected by lateral channels. Based on the micro-computed tomography results, no more than three canal anastomoses were detected in all teeth. Hence, three lateral channels were created in the canal anastomosis model. The experimental setup (Fig. 2b–f) is schematically represented in Fig. 3 for clarification of the fluid inflow-outflow and pressure signal recording logistics.

The two models were used for repeated measurements of fluid pressure generated in the periapical region, when 2% NaOCl was delivered at different fluid flow rates and needle insertion depths. The experimental setup represented a closed canal design which did not allow fluid to be extruded through the faux apical termination, thereby allowing periapical pressures to be recorded via pressure sensors. Two needle insertion depths were employed, with the needle positioned at 1 or 3 mm short of the faux apical termination^7, 11^. With the use of a 30-gauge side-venting needle, flow rates of 1.5 mL/min (0.025 mL/sec) and 15.6 mL/min (0.26 mL/sec) were used to represent the low-end^26^ and high-end^11^ of clinically relevant irrigant delivery rates, respectively. To achieve a flow rate higher than 15 mL/min, the 5 mL syringe described in the Experimental Setup required modification. Understandably, clinically relevant fluid flow rates may vary between these two opposing ends of the spectrum. A pilot study was thus performed, using flow rates from 1–16 mL/min and with incremental increase of 1.0 mL/min (0.17 mL/sec), to determine the sample size and to identify the lowest fluid flow rate for 4 kPa fluid pressure to be generated in each canal model. The rationale for the use of the 4 kPa cut-off was that the pressure represents the average intraosseous pressure of the sinusoids in cancellous bone^27^. Based on the results derived from the pilot study, a fluid flow rate of 2.5 mL/min (0.042 mL/sec) was identified as the flow rate beyond which periapical pressures exceeding 4 kPa could be generated when the side-vented needle was inserted to 1 mm above the apical terminus in the separate canal model (Supplementary Fig. S1-a). Likewise, a fluid flow rate of 9.5 mL/min (0.158 mL/sec) was identified as the flow rate beyond which periapical pressures exceeding 4 kPa could be generated when the side-vented needle was inserted to 1 mm coronal to the apical terminus in the canal anastomosis model (Supplementary Fig. S1-b). Regression analyses indicate that for both models, significant relations exist between fluid flow rate and periapical pressure that can be represented by second-order polynomial equations. For examining fluid pressures in 16 groups (2 canal models × 2 needle insertion depths × 4 fluid flow rates), sample size analysis (α = 0.05; desired power = 0.8) indicated that 12 recordings/group were necessary for identification of a minimum detectable difference of 1.6 kPa in the means of the generated periapical pressure, for an anticipated standard deviation of 0.867 kPa.

**Figure 4:**
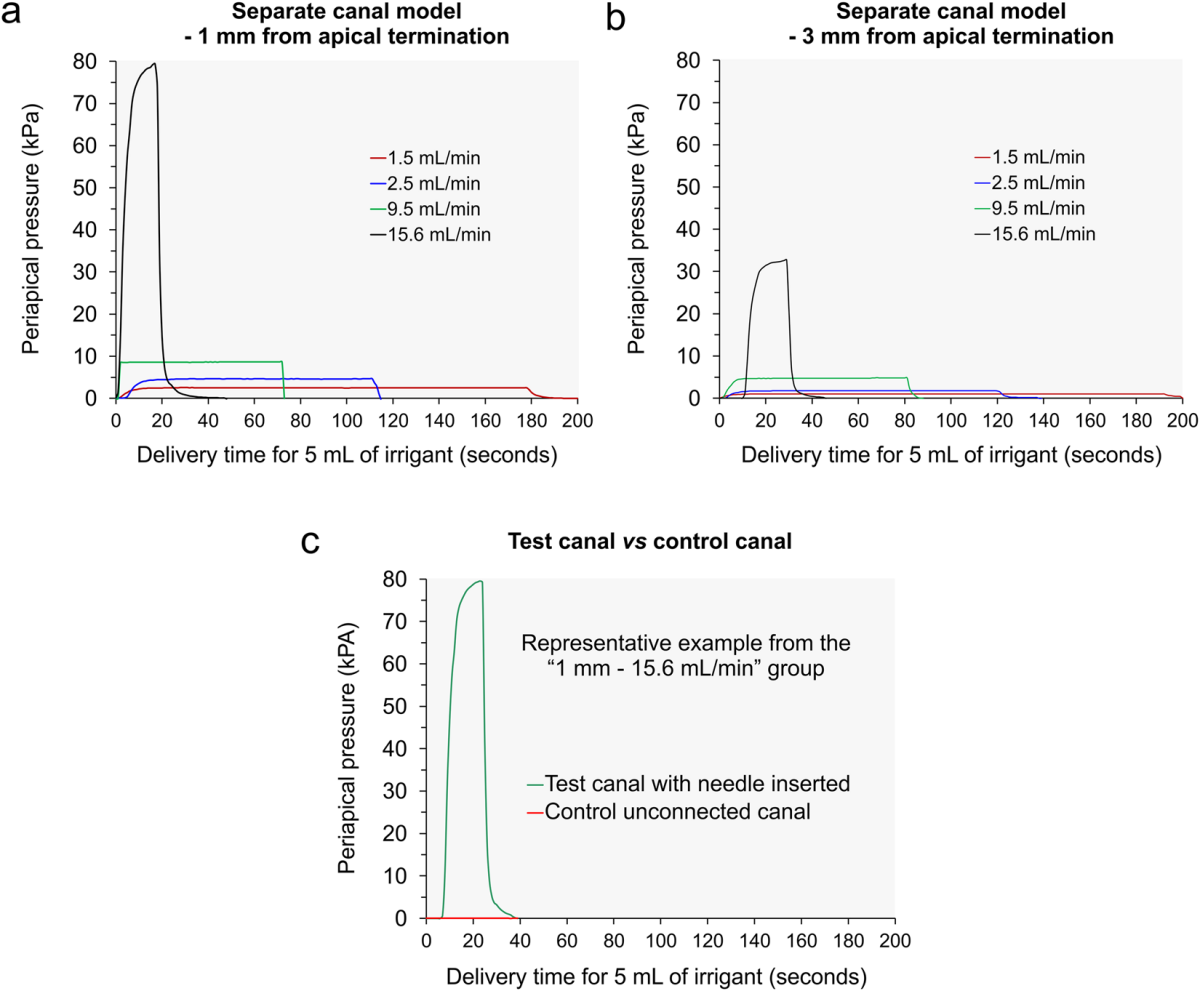
Apical fluid pressures generated in the separate canal model with different fluid flow rates. (**a**) Representative pressure-time plots produced by inserting the side-venting needle to 1 mm above the faux apical terminus in the test canal. (**b**) Representative pressure-time plots produced by inserting the side-venting needle to 3 mm above the faux apical terminus in the test canal. (**c**) Representative example of a superimposed pressure-time plot produced in one recording of the test canal and the control unconnected canal.

Figure 4 features pressure-time plots generated from the separate canal model. Representative plots produced by inserting the side-venting needle to 1 mm above the faux apical terminus in the test canal are shown in Fig. 4a. Plots produced using the flow rates of 1.5, 2.5, 9.5 and 15.6 mL/min for delivering 5 mL of NaOCl were superimposed together to provide an appreciation of the differences in the time required for delivery of the same volume of irrigant at those designated flow rates. In addition, the same scale was employed for the ordinate axis to illustrate the differences in the magnitude of fluid pressure generated at these fluid flow rates. Similarly, representative plots produced by inserting the side-venting needle to 3 mm above the faux apical terminus in the test canal are shown in Fig. 4b. Because these plots were prepared using the ordinate axis scale as Fig. 4a, the reduction in maximum fluid pressure produced by raising the side-vented needle for just 2 mm for each fluid flow rate may be readily appreciated. Figure 4c is a representative example illustrating the pressure difference in a test canal containing the side-vented needle and the adjacent needle-free control canal. For all needle insertion depths and fluid flow rates, pressures generated in the control canal were close to null pressure (±0.0067 kPa). The null pressure values recorded in the control canal attested that a fluid-tight seal was achieved by the machinist set-up clamps in clamping the two block-halves to form the simulated root canal (see Methods).

**Figure 5:**
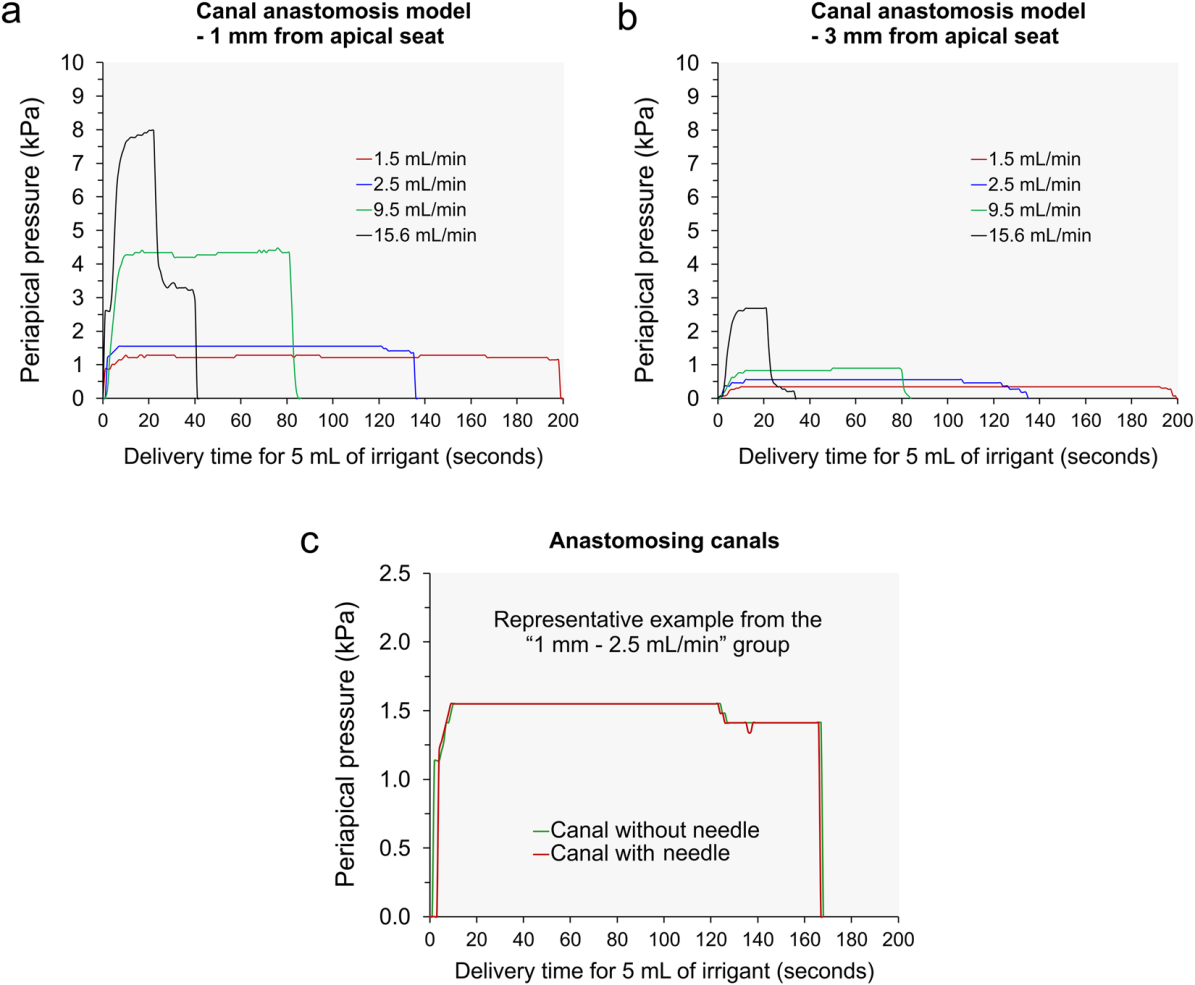
Apical fluid pressures generated in the canal anastomosis model with different fluid flow rates. (**a**) Representative pressure-time plots produced by inserting the side-venting needle to 1 mm above the faux apical terminus in the test canal. (**b**) Representative pressure-time plots produced by inserting the side-venting needle to 3 mm above the faux apical terminus in the test canal. (**c)** Representative example of a superimposed pressure-time plot produced in one recording of the test canal and the control connected canal.

Figure 5 represents pressure-time plots generated from the canal anastomosis model. Representative plots produced by expressing NaOCl with different flow rates through the side-venting needle inserted to 1 mm above the apical terminus in the test canal are shown in Fig. 5a. Representative plots produced by inserting the side-venting needle to 3 mm above the apical termination are shown in Fig. 5b. Note that the scale of the ordinate axis in these two charts was only one-eighth of that in Fig. 4a and b, because of the extensive reduction in the maximum periapical fluid pressure generated in the test canal. Because the two parallel canals were connected, the periapical pressures generated in these canals were very similar (±0.0067 kPa). This is illustrated by the superimposition of the pressure-time plots obtained from the test canal and the adjacent canal during a single measurement (Fig. 5c). The principle behind such an observation is didactically conveyed by the Pascal’s law of hydrostatics, which states that pressure applied on a confined incompressible fluid (i.e. density does not change) is transmitted in all directions with equal force on equal areas^28^.

**Figure 6:**
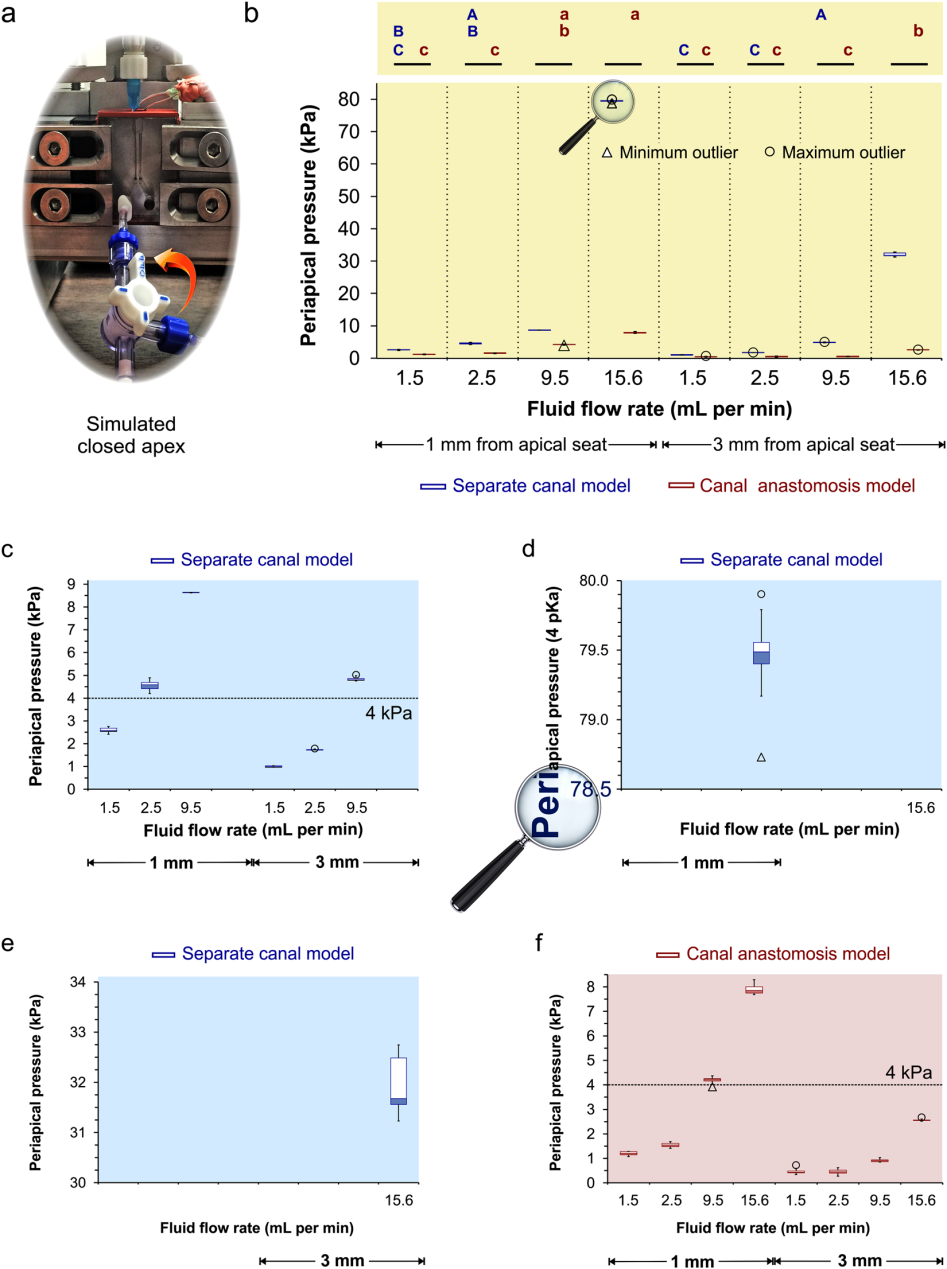
Data analysis. (**a**) A closed-canal system was produced in the experimental models to enable detection of periapical pressure build-ups by the pressure sensors. (**b**) Overview of box-and-whisker plots of pressures generated by the two models. For pairwise comparisons within the separate canal model, groups identified with the same upper-case letters are not significant different (p > 0.05). For pairwise comparisons within the canal anastomosis model, groups identified with the same lower case letters are not significant different (p > 0.05). For inter-model comparisons, groups identified with a green bar are significant different (p < 0.05). (**c**) High magnification of the separate canal model plots for 1.5, 2.5 and 9.5 mL/min fluid flow rates at 1 and 3 mm from the apical termination. Values are medians and quartiles. The dotted line at 30 mm Hg represents intraosseous space blood pressure. (**d**) High magnification of the separate canal model plot for 15.6 mL/min at 1 mm from the apical terminus. (**e**) High magnification of the separate canal model plot for 15.6 mL/min at 3 mm from the apical terminus. (**f**) High magnification of the box-and-whisker plots in the canal anastomosis model.

The maximum apical pressure detected from the test canals in the 16 groups are collectively represented in Table 1. Because the data were not normally distributed and heteroscedastic even after non-linear transformations, the values are expressed as medians and interquartile ranges. Results of the non-parametric statistical analyses are shown as box-and-whisker plots in Fig. 6a. These plots represent variations in a statistical population without making assumptions of the underlying statistical distribution. High magnifications of individual box-and-whisker plots are shown in Fig. 6c–f for better visualisation of the first and third quartiles of each data set.

**Table 1:** Maximum apical fluid pressures generated from **A**. the separate canal model and **B**. the canal anastomosis model, by irrigant delivered at different flow rates (in mL/min) following the insertion of a side-vented needle to different depths (in mm) from the simulated apical termination of the tested canal. Data for the adjacent canal are not shown. Values represent medians (*interquartile range*).

**1 mm** from simulated apical termination				**3 mm** from simulated apical termination			
**Fluid flow rate** (mL/min)				**Fluid flow rate (**mL/min)			
**1.5**	**2.5**	**9.5**	**15.6**	**1.5**	**2.5**	**9.5**	**15.6**
**A**							
**Separate canal model** (**N** = **12**; **kPa**)							
2.59	4.58	8.63	79.49	1.00	1.72	4.81	31.68
(*0.16*)	(*0.28*)	(*0.01*)	(*0.16*)	*(0.07*)	(*0.02*)	(*0.08*)	(*0.92*)
**B**							
**Canal anastomosis model** (**N** = **12**; **kPa**)							
1.21	1.55	4.23	7.85	0.43	0.45	0.90	2.55
(*0.14*)	(*0.13*)	(*0.09*)	(*0.26*)	(*0.07*)	(*0.13*)	(*0.08*)	(*0.03*)

Statistical analysis of the data derived from the separate canal model, using repeated measures analysis of variance (ANOVA) on ranks, indicated that the differences in median values among the 8 groups (2 needle insertion depths × 4 fluid flow rates) were greater than would be expected by chance; there was a statistically significant difference among those groups (p ≤ 0.001). Pairwise comparisons using the Dunn’s method (Fig. 6b) indicated that periapical pressure acquired from the 4^th^ group (1 mm from apical termination – 15.6 mL/min, represented as 1/15.6 thereafter) was significantly higher (p < 0.05) than the 8^th^ group (3/15.6), which, in turn was significantly higher than the 3^rd^ group (1/9.5). Periapical pressures derived from these three groups were all significantly higher than the other five groups. There was no significant difference in the maximum pressure generated by the 7^th^ group (3/9.5) and the 2^nd^ group (1/2.5), and between the pressures generated by the 2^nd^ group and the 1^st^ group (1/1.5). Pressures generated by the 1^st^ group, 5^th^ group (3/1.5) and the 6^th^ group (3/2.5) were not different from one another, and were significantly lower than the pressures generated by the other 5 groups. Likewise, analysis of the data derived from the canal anastomosis model, using repeated-measures ANOVA on ranks, detected a statistically significant difference among the 8 groups (p < 0.001). Pairwise comparisons (Fig. 6b) indicated that the maximum apical pressures were in the descending order: 4^th^ group (1/15.6) = 3^rd^ group (1/9.5) > 3^rd^ group = 8^th^ group (3/15.6) > 2^nd^ group (1/2.5) = 1^st^ group (1/1/5) = 7^th^ group (3/9.5) = 5^th^ group (3/1.5) = 6^th^ group (3/2.5). For inter-model comparison using individual Mann-Whitney tests, a statistically significant difference (p < 0.05) was identified for each of the 8 parameter combinations (Fig. 6b).

Previous studies reported that both increased pressure during irrigant delivery and a high irrigant flow rate may cause irrigant extrusion through the root apex into the periapical regions^29–32^. However, the relationship between fluid pressure and fluid flow rate has not been clarified. From Supplementary Fig. S1-a, it can be seen that the relationship between fluid flow rate and periapical pressure is non-linear. For incompressible, steady, inviscid flow, the Bernoulli equation of energy conservation for flowing fluids states that the sum of all forms of energy in a fluid along a streamline (in the form of static pressure, dynamic pressure and hydrostatic pressure) remains the same at all points along that streamline^28^. Although water is incompressible, the Bernoulli equation should be used with reservation in accounting for fluid flow in the present situation because of potential violation of the assumptions of an inviscid fluid and steady flow characteristics. Compared with liquid helium, water is a fairly viscous fluid because the water molecules are attracted to each other by hydrogen bonds. Because effort is required to push the attracted molecules aside, there is irreversible energy loss caused by friction along the canal wall boundary during fluid movement^8^. The energy loss is dependent upon the Reynolds number characteristic of the velocity and the dynamic viscosity of the fluid within the system. In computational fluid dynamics simulation of needle irrigation of root canals, the fluid flow characteristic also changes from laminar to turbulent flow with increasing fluid velocities^33^. Notwithstanding these limitations, what the pressure sensor detects is predominantly the dynamic pressure generated by the influxing fluid head; contributions from the static pressure and hydrostatic pressure are minimal by comparison. According to Newton’s second law, this dynamic pressure is equivalent to the kinetic energy of the moving fluid mass, and is represented by 0.5 m*v* ^2^, where m is the fluid mass and *v* is the effective fluid velocity^28^. The latter is the quotient obtained by dividing fluid flow rate (Q) with the net surface area (A) available for fluid flow (i.e. *v* = Q/A). Since the net surface area remains the same when the needle tip is fixed at a designated insertion depth, it may de deduced that the dynamic pressure generated is related to the square of the fluid velocity (*v* ^2^) and hence the square of the fluid flow rate (Q^2^).

Apart from fluid flow rate, dynamic pressures detected by the pressure sensor also vary with needle insertion depth. Using the separated canal model and a fixed fluid flow rate of 10 mL/min in another pilot study, the dynamic pressures generated by inserting the needle to 1–10 mm coronal to the apical terminus were recorded in 0.5 mm increments, while keeping the side-vent outlet in the same orientation. The relationship between the recorded pressures and needle insertion depth is also non-linear (Supplementary Fig. S2, experimental portion). At a designated needle insertion depth, the net surface area available for fluid flow, as mentioned is the previous paragraph, refers to the difference between the cross-sectional areas of the 0.04 taper canal and the external needle surface. The net canal cross-sectional surface areas available for fluid flow when a 30-gauge needle is inserted to 1–10 mm from the faux apical terminus are depicted in Supplementary Table S1. Because pressure equals force divided by surface area, the force used to produce a flow rate of 10 mL/min was derived from the experimental data. This force value was then employed for calculating the theoretical pressure produced by fluid passing through the respective net surface area (Supplementary Table S1). When the theoretically deduced pressure values were plotted against the corresponding needle insertion depths (Supplementary Fig. S2, theoretical deduction portion), the resulting plot was similar to plot of actual measured pressure values against needle insertion depths. The theoretical deductions at insertion depths further away from the apical terminus were generally higher than the actual experimental values. This may be caused by irreversible frictional head loss (i.e. Darcy-Weisbach frictional factor) that was not taken into account in the theoretical estimations^28^. Nevertheless, the similarity of the two plots indicates that the dynamic pressure recorded at a designated insertion depth is a function of the net surface area available for fluid flow. The irrigant expressed ahead of the side-vent must flow coronally and bypass the partially-occluded space created by the irrigation needle. The needle decreases the cross-sectional area of the canal, creating a bottle-neck that increases the back pressure in the region apical to the irrigation needle. The continuity equation for fluid dynamics states that mass is conserved when a fluid is in motion^28^. That is, under the assumption of steady flow, the product of the surface area (A) available for fluid flow and the fluid velocity (*v*) remains constant (A_1_ *v*_1_ = A_2_ *v*_2_). Thus, for a constant fluid flow rate, increase in net surface area is associated with a corresponding decrease in fluid velocity. Accordingly, the further the needle is away from the apical terminus, the lower will be the periapical pressure, due to reduction in kinetic energy of the fluid mass (0.5 m*v* ^2^).

The relationship between net surface area available for fluid flow and periapical pressure has important clinical implications. It has been previously reported that for a 30-gauge side-venting needle, the advancing front of an irrigant expressed from the outlet of the side-vent is limited to 1–1.5 mm, beyond which efficacious debridement cannot be achieved^33^. The results of the present study indicate that in the presence of high fluid flow rates, pressures exceeding the average human intraosseous pressure (4 kPa) may be produced even when the needle tip is more than 1 mm away from the apical terminus. Such a scenario may occur during debridement of a highly calcified canal, wherein the final shape of the instrumented canal closely approximates the geometry of the rotary instruments used for canal preparation. By contrast, chemical debridement of a single oval-shaped, non-calcified root canal is less taxing. From a pressure generation perspective, increase in the net surface area contributed by canal fins and cul-de-sacs is analogous to moving the needle further away from the apical terminus. Another “invisible” element that should be brought to attention is that, unlike polycarbonate, dentinal tubules are present in the intraradicular dentine. Patent dentinal tubules contribute to an “invisible” increase in surface area for extra pressure reduction. However, even when non-sclerotic dentinal tubules are present in the apical third of the canal space, they are packed with smear plugs immediately after mechanical instrumentation. These dentinal tubules are only rendered patent following irrigant-dependent dissolution of the inorganic and organic components of the smear layer. Hence, in theory, a root canal is most vulnerable for intracanal pressure build-up immediately following canal instrumentation. This point will be elaborated further in the discussion of the results derived from the canal anastomosis model.

In the presence of communicating channels in the canal anastomosis model, the magnitude of periapical pressure build-up decreased by almost 90% irrespective of the fluid flow rate or needle insertion depth. Thus, the null hypothesis tested in the present study has to be rejected. Reduction in periapical pressures in anastomoses-containing roots provides an explanation why pressure generation in single canals is considerably higher. Although the likelihood of raising the intracanal fluid pressure to beyond the intraosseous pressure is considerably more remote in canals connected by isthmi and/or lateral channels, one can see from Fig. 6f that this is still possible when the needle tip is close to the apical terminus and when the fluid flow rate is sufficiently high.

The presence of communicating channels in the canal anastomosis model is analogous to the installation of pressure relief valves in a hydraulic circuit. These channels provide the path of least resistance for the incoming fluid to be diverted from the canal being irrigated to the second canal. Because there is less fluid moving toward the apical region, the pressure sensor detects less kinetic energy from the reduced mass of moving fluid. The extent of fluid diversion is likely to be influenced by the number of channels, the size of the channels and “loss of the channels”. In general, loss of a channel may be due to “frictional loss”, which is Reynolds number dependent, or “form loss”, which is geometry dependent, both of which have to be determined empirically. Although dentine is rigid and cannot expand or contract to alter the anatomical dimension of the bypass channels, a clinically relevant example of “frictional and form loss” may be caused by encroachment of the lateral channels by pulpal tissues, debris from mechanical preparation, or retained temporary dressings such as calcium hydroxide. These materials can decrease the functional dimensions of the channels and increase friction during fluid movement, thereby reducing the efficacy of fluid diversion. A similar reduction in anatomic diameter occurs when dentinal tubules contain intratubular materials such as collagen; decrease in functional diameter of the dentinal tubules causes substantial reduction in the hydraulic conductance of dentine^34^.

In the present study, the side-vent outlet was oriented 180° from the bypass channels during all pressure measurements (see Methods). Paradoxically, we found that even with the use of a fixed flow rate and a fixed needle insertion depth, the recorded pressures may vary substantially with the orientation of the side-vent outlet. This is illustrated by the results of a preliminary study comparing the periapical pressures recorded in the canal anastomosis model, with the side-vent facing the communicating channels or oriented 180° from the channels (Supplementary Fig. S3). Compared with the 180° orientation, periapical pressures are further reduced when the side-vent outlet is facing the channels directly. In the context of fluid diversion, the jet of irrigant that extruded from the side-vent outlet is directed toward the apical terminus with a divergence of approximately 30 degrees (reference). If the outlet is oriented 180° away from the communicating channels, the jet of irrigant is preventing the irrigant from flowing backwards into the area of the side-vent and forces the irrigant apically and around the needle tip. This will increase the resistance to fluid flow and diversion of the irrigant into the adjacent canal and hence increasing the periapical pressure. Conversely, if the outlet is facing the communicating canals directly, the jet of irrigant can be diverted into the other canal without much resistance, thereby reducing the periapical pressure even further. While this issue is of academic interest, it has relatively little clinic significance because it is impractical to ensure that the side-vent outlet of a needle is facing the openings of the canal anastomoses every time a needle is inserted into a root canal. Moreover, without the use of a contrasting medium (such as the use of radiopaque calcium hydroxide), it is impossible to determine that anastomoses exist between two adjacent canals even with the use of cone-beam computed tomography.

The use of a polycarbonate model to simulate root canals and anastomoses in the present study does have limitations. The described model does not represent all or even part of normal clinical canal morphology but does provide a means for examining the effect of intracanal anastomosis on apically directed pressure. The results of the present work should be repeated in the future using multiple measurements of the fluid pressure profiles in real teeth. Because canal anastomoses and canal morphology are highly heterogenic, it is necessary for studies involving the use of biological specimens to include a sufficient number of specimens and use randomisation to minimise “unwanted noise”. The data should be more robust in validating the hypothesis and the conclusion derived should be more clinically relevant.

## Conclusion

Within the limits of the present *in vitro* study using polycarbonate block models to simulate natural teeth with and without canal anastomoses, it may be concluded that pressure reduction in anastomoses-containing roots provides an explanation why pressure generation in single roots is considerably higher. From a clinical perspective, root canals with a highly variable degree of anastomoses may be expected in even single-rooted teeth with double canals. Unfortunately, these anastomoses are not readily recognisable with periapical radiographs or cone-beam computed tomography. Hence, clinicians have to use caution in irrigating root canals to prevent inadvertent pressure build-up. It is known from a previous study that the intraosseous pressure is smaller than 4 kPa^12^. The results of the present work indicate that a clinician can stay in the safe zone of preventing the generation of a potential NaOCl accident when the irrigant is delivered at a rate of lower than 4 mL/min in all situations, including conservatively-shaped canals, or canal systems with complex anastomoses. It is important for clinicians to appreciate that it is still possible in teeth with canal anastomoses for pressure exceeding the intraosseous pressure to be generated when the fluid flow rate is sufficiently high and when the needle tip is close to the apical terminus.

## Methods

### Development of root canal models

Two hundred extracted premolars with completely formed apices from a native Chinese population were randomly selected from the tooth and canal morphology database (School of Stomatology, Wuhan University, China) for micro-computed tomography. The premolars were scanned (μCT 50, Scanco Medical, Bassersdorf, Switzerland) with a nominal isotropic resolution of 15 μm inside a 48-mm diameter scanning vial at 90 kVp, 88 mA, 8 W, 500 msec integration time and 500 projections per 180°. One hundred and fifty-three premolars with singled-rooted roots were detected, of which 84 teeth had double canals. Three-dimensional representations of each root canal system was rendered with VGStudio MAX 2.1 (Volume Graphics, Heidelberg, Germany).

### Model construction

The models were each constructed from two separate blocks of impact-resistant polycarbonate (McMaster-Carr, Santa Fe Springs, CA, USA). For the separate canal model (Fig. 2a, left), each block was lapped with 2000-grit silicon carbide sheets (McMaster-Carr) on top of a granite flat-surface polishing block until each surface was highly-polished and free of visible scratches. The blocks were then clamped together and indexing pin-holes (McMaster-Carr) were machined in the opposing corners. Standard size 2–56 flat-head were subsequently machined in the other corners that produced a single combined block when the two halves were pinned and screwed together. The front-facing half of the combined block was drilled and reamed to the dimensions of a standard female Luer hole so that a male Luer fitting could be subsequently inserted. This hole extended completely through the front plate and 2 mm into the back plate. The blocks were then disassembled and two 1.0 mm holes were drilled to a depth of 2.0 mm in the back plate such that the top of each hole was 23 mm from the top of the block, with a separation of 1.0 mm between the holes. At this point, the back block contained two separate faux apical terminations and two extended holes from the front plate containing the Luer holes. The two areas were then connected by a channel shown in the apical area of Fig. 2a, enabling each faux apical termination to be connected directly to the female Luer hole. After reassembling the two halves, a 2.0 mm wide access opening was created (Fig. 2a, top) and two parallel holes (0.34 mm in diameter and 2.0 mm centre-to-centre) were used to connect the access opening to the individual faux apical termination. The final canal shape was completed by instrumenting the entire length with a size 30, 0.04 taper hand file until the tip of the instrument just cleared the top of the faux apical termination.

The final separate canal model consisted of two separate simulated instrumented root canals: 1) the test canal on the left (Fig. 2a, solid long arrow) for inserting a 30-gauge closed-end side-venting needle (Max-i-Probe, Dentsply Sirona, York, PA, USA) that remained non-binding within the canal space following its insertion to the designated depth, and 2) the control, unconnected canal on the right (Fig. 2a, open arrow) for recording pressure changes in that canal in the absence of needle insertion. The aforementioned procedures were repeated for the canal anastomosis model (Fig. 2a, right). On completion, one block was disassembled and three lateral anastomosis channels were prepared to connect the two simulated root canals (Fig. 2a, open arrowhead). The blocks were then reassembled (Fig. 2b). Those anastomosis channels were shaped with a 0.5 mm diameter machinist mill cutter so that their sizes were 0.5 mm wide and deep.

### Experimental setup

Because irrigant flow rate and the volume of irrigant delivered were critical variables in the present study, a programmable precision syringe pump (Fig. 2b-a; Legato 100 SNGL SYR PUMP, World Precision Instruments, Sarasota, Fl, USA) was used to control those variables. A 5 mL syringe was placed in the pump (Fig. 2b-b) for the delivery of 2% NaOCl as the irrigant. The 5 mL syringe required a custom-made sheath so that it could withstand the force to generate flow rates higher than the flow rate of 15 mL/min described in the literature^11, 31^; clinically, these rates are only manually possible by using a 1 mL syringe. To ensure delivery of the exact volume of irrigant, no air bubbles were allowed to enter any part of the delivery system. This criterion was fulfilled by using a 50 mL syringe (Fig. 2b–d) connected to a two-way valve to refill the 5 mL delivery syringe. Using the refill syringe with its tip pointed down caused all air bubbles to rise to the plunger, thereby preventing air injection into the delivery system.

The test unit shown at the right side of Fig. 2b accomplished several objectives: First, it served to hold the test model in a stationary position while forcing them together with machinist set-up clamps (McMaster-Carr, Santa Fe Springs, CA; Fig. 2b, asterisk) to prevent irrigant from escaping from the simulated root canal system. Second, the test delivery needle was held in place by a positioning head that was free to move up and down and the height of which could be controlled with precision by using machine gauge blocks. Briefly, a 40-mm thick metal block (Fig. 2b-b) was first set under the positioning head that held a 9.525 mm-diameter, hollow aluminum rod in position via a set-screw. This rod was machined to receive a male Luer fitting at the top and a female Luer fitting at the bottom. With the movable holding head resting on the 40-mm thick metal block, the delivery needle was moved to full working length. For the canal anastomosis model, the needle was inserted with the side-vent outlet oriented at 180° away from the lateral communications. This orientation was maintained during all subsequent recordings in both models. Additional metal gauge blocks of different thickness were subsequently stacked on top of the 40 mm-thick block, so that the needle tip could be repositioned, without binding, to different depths above the faux apical termination. The polyvinyl chloride (PVC) tubing exiting the syringe pump was attached directly to the aluminum positioning rod. Third, an independent aluminum block was used to hold a blunt needle very close (Fig. 2c, solid arrowhead) to the access opening of each model to be tested. This blunt needle, connected to the fluid evacuation system of a dental operatory unit, prevented irrigant overflow by aspirating excess irrigant exiting the canal access opening.

### Pressure determination

A schematic of the fluid inflow-outflow and pressure signal recording in the test system is shown in Fig. 3. Each pressure sensor (Fig. 2d; PREPS-N-000, PendoTech, Princeton, NJ, USA) was protected by an in-line Luer fitting. This setup enabled the sensor to be connected via PVC tubing (Fig. 2c, arrow) to a Luer port placed in the front of the canal split-block. A Luer on-off valve (Fig. 6a) was inserted between each pressure sensor and the Luer port. Opening the on-off valve enabled the tubing and the artificial root canal system to be filled with an incompressible, air bubble-free liquid (i.e. the NaOCl irrigant) via the “primer syringe”. After filling with irrigant, the on-off valve was closed (arrow in Fig. 6a) to generate a closed canal system (i.e. a canal system that does not permit pressure relief via extrusion of the irrigant into the periapical spaces). Once the system was “primed” to create a close canal system, pressure changes in the needle-containing test canal and the adjacent canal (without needle) could be recorded. Signals were generated by the pressure channels in millivolts at one-second intervals. The pressure sensors were connected via cables (Fig. 2b,c) to a signal-conditioning unit (Fig. 2e; Model PDKTP-FP, PendoTECH), to convert the millivolt analogue signals generated by the pressure sensors to electronic digital signals. Signals generated from the test canal and adjacent parallel canal were recorded simultaneously by separate channels in the signal-conditioning unit. The digital signals were transmitted via a Universal Serial Bus cable to a laptop computer that recorded the pressure changes at 1 Hz frequency using proprietary software supplied by the manufacturer. Data derived from the 16 groups were saved as Excel files (MicroSoft Corp., Redmond, WA, USA). Apical fluid pressure data, recorded in pounds per square inch, were converted into mm Hg and plotted as pressure-time plots. The highest pressure recorded by a channel in each pressure-time plot was used to present the maximum periapical fluid pressure produced in each canal.

### Statistical analyses

A three-factor analysis of variance (ANOVA) was initially planned to examine the effects of the ability for collateral redirection of an incompressible fluid (separate canal model *vs* canal anastomosis model), needle insertion depth (1 mm *vs* 3 mm from the simulated apical seat) and irrigant flow rate (1.5, 2.5, 9.6 and 15.6 mL/min) on the maximum periapical pressure generated in the test canals. However, the normality and equal variance assumptions of the data sets were violated even after nonlinear transformations. Hence, data sets from the separate canal model (2 needle insertion depths × 4 fluid flow rates) were analysed with repeated measures ANOVA on ranks and Dunn’s multiple comparison tests, taking into consideration that measurements were repeatedly recorded within the same experimental setup. Likewise, data sets from the separate canal model (2 needle insertion depths × 4 flow rates) were analysed with repeated measures ANOVA on ranks and Dunn’s multiple comparison tests. For comparison between the separate canal model and the canal anastomosis model, the two data sets generated using a particular needle insertion depth and fluid flow rate were analyzed with the Mann-Whitney rank sum test. For all analyses, statistical significance was pre-set at α = 0.05.

## Electronic supplementary material

Supplementary Information

Supplementary Video S-1a

Supplementary Video S-1b

Supplementary Video S-1c

Supplementary Video S-1d
