# Supplementary material for: Effect of Canal Anastomosis on Periapical Fluid Pressure Build-up during Needle Irrigation in Single Roots with Double Canals using a Polycarbonate Model
Source: Sci Rep. 2017 May 8;7:1582. doi: 10.1038/s41598-017-01697-1 (PMC5431501; doi:10.1038/s41598-017-01697-1)
Supplement: Supplementary file 1 — Supplementary Information [file 41598_2017_1697_MOESM1_ESM.pdf]

Effect of Canal Anastomosis on Periapical Fluid Pressure Buildup during Needle Irrigation in Single Roots with Double Canals Using a Polycarbonate Model

Qi Huang[a†], Jonathan B. Barnes[b†], G. John Schoeffel[c], Fan Bing[d], Candice Tay[e], Brian E. Bergeron[b], Lisiane F. Susin[a], Jun-qi Ling[a] *, Li-na Niu[f] *, Franklin R. Tay[a] *

[†]Equal contributors

*Corresponding authors

Supplementary Information

1. Figure S-1
2. Table S-1
3. Figure S-2
4. Figure S-3
5. Supplementary Videos S-1a, S1-b, S1-c and S1-d corresponding to the microcomputed tomography images shown in Figure 1a-1d of the main text.

Supplementary Figure S-1

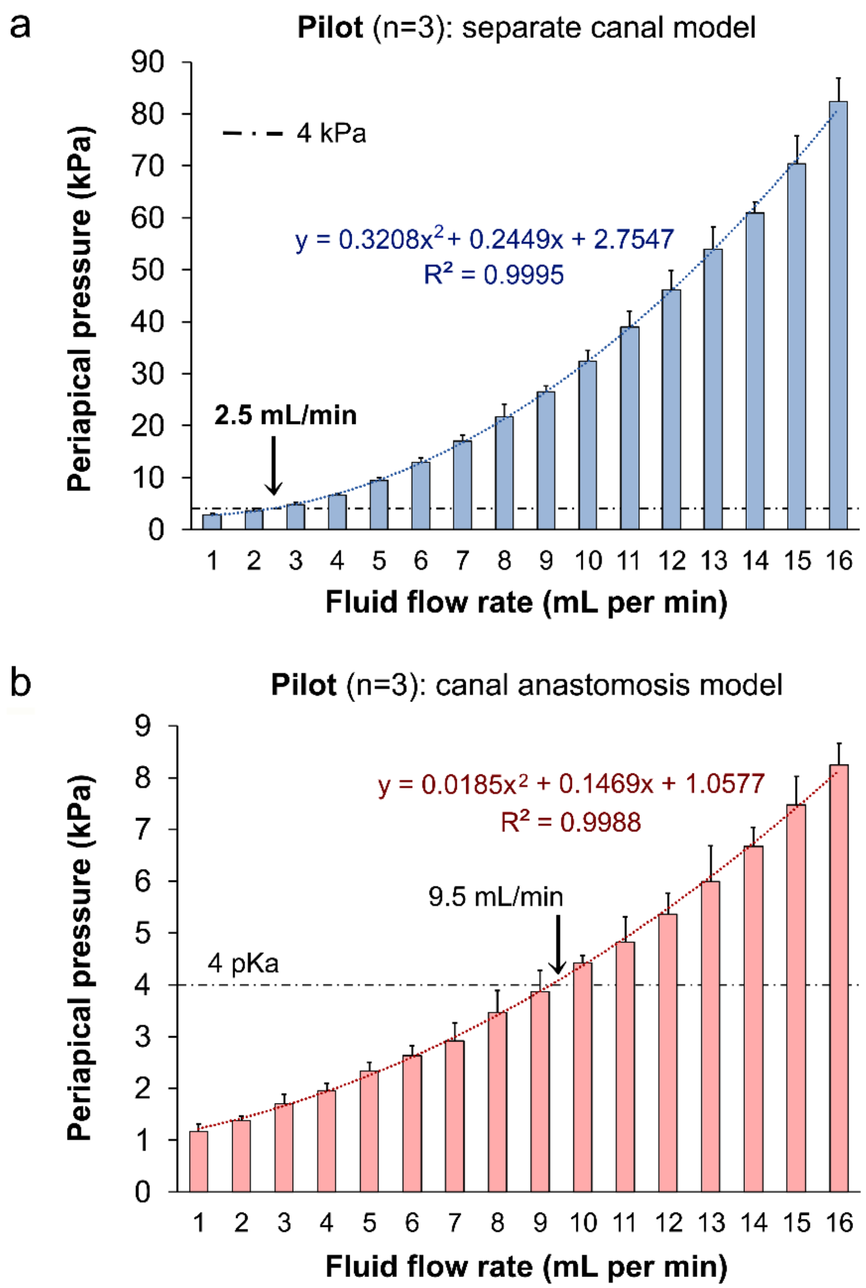

Figure S-1 Periapical pressures recorded in **a**. the separated canal model, and **b**. the canal anastomosis model, using incremental fluid flow rates from 1-16 mL/min. For all measurements, the depth of insertion of the needle tip was maintained at 1 mm short of the faux apical terminus, and the side-vent of the needle was oriented facing the lateral channels in the second model.

Table S-1 Measured and theoretically deduced periapical pressures obtained by inserting the irrigant delivering needle to different depths in the separated canal model with irrigant delivered at a flow rate of 10 mL/min.

Distance of needle tip from faux apical terminus (mm)	**Periapical pressure (measured)** (kPa)	Canal diameter (0.04 taper) (m)	Canal area (m^2)	Needle outer surface area (m^2)	Net canal area (m^2)	Force produced at 10 mL/min (kgf)	**Periapical pressure (theoretical deduction)** (kPa)
1.0	**41.92**	3.9 E-4	1.19 E-07	8.00 E-08	3.95 E-08	1.654 E-06 *	41.92
1.5	**33.16**	4.1 E-4	1.32 E-07		5.20 E-08		31.79
2.0	**29.58**	4.3 E-4	1.45 E-07		6.52 E-08		25.36
2.5	**23.99**	4.5 E-4	1.59 E-07		7.90 E-08		20.93
3.0	**16.27**	4.7 E-4	1.73 E-07		9.35 E-08		17.96
3.5	**11.51**	4.9 E-4	1.89 E-07		1.09 E-07		15.23
4.0	**10.13**	5.1 E-4	2.04 E-07		1.24 E-07		13.31
4.5	**8.41**	5.3 E-4	2.21 E-07		1.41 E-07		11.76
5.0	**6.76**	5.5 E-4	2.38 E-07		1.58 E-07		10.50
5.5	**6.20**	5.7 E-4	2.55 E-07		1.75 E-07		9.44
6.0	**6.14**	5.9 E-4	2.73 E-07		1.93 E-07		8.55
6.5	**6.00**	6.1 E-4	2.92 E-07		2.12 E-07		7.79
7.0	**5.24**	6.3 E-4	3.12 E-07		2.32 E-07		7.14
7.5	**4.89**	6.5 E-4	3.32 E-07		2.52 E-07		6.57
8.0	**4.69**	6.7 E-4	3.53 E-07		2.73 E-07		6.07
8.5	**4.55**	6.9 E-4	3.74 E-07		2.94 E-07		5.63
9.0	**4.00**	7.1 E-4	3.96 E-07		3.16 E-07		5.24
9.5	**3.72**	7.3 E-4	4.19 E-07		3.39 E-07		4.89
10.0	**3.52**	7.5 E-4	4.42 E-07		3.62 E-07		4.57

* Derived from force = pressure x area, using measured pressure and net canal area at 1.0 mm from the apical terminus

Supplementary Figure S-2

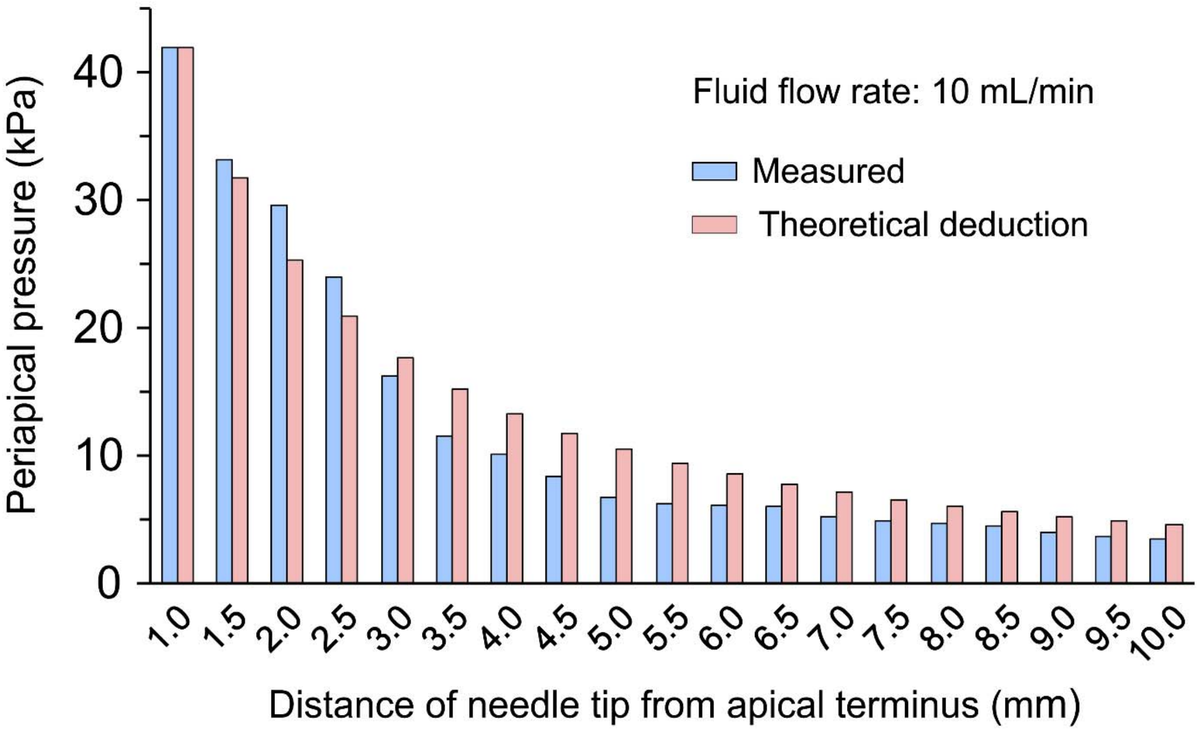

Figure S-2 Graphic representation of the measured and theoretically deducted periapical pressures (data from Table S-1) obtained with an irrigant flow rate of 10 mL/min, after inserting the needle tip to different depths in the separated canal model.

Supplementary Figure S-3

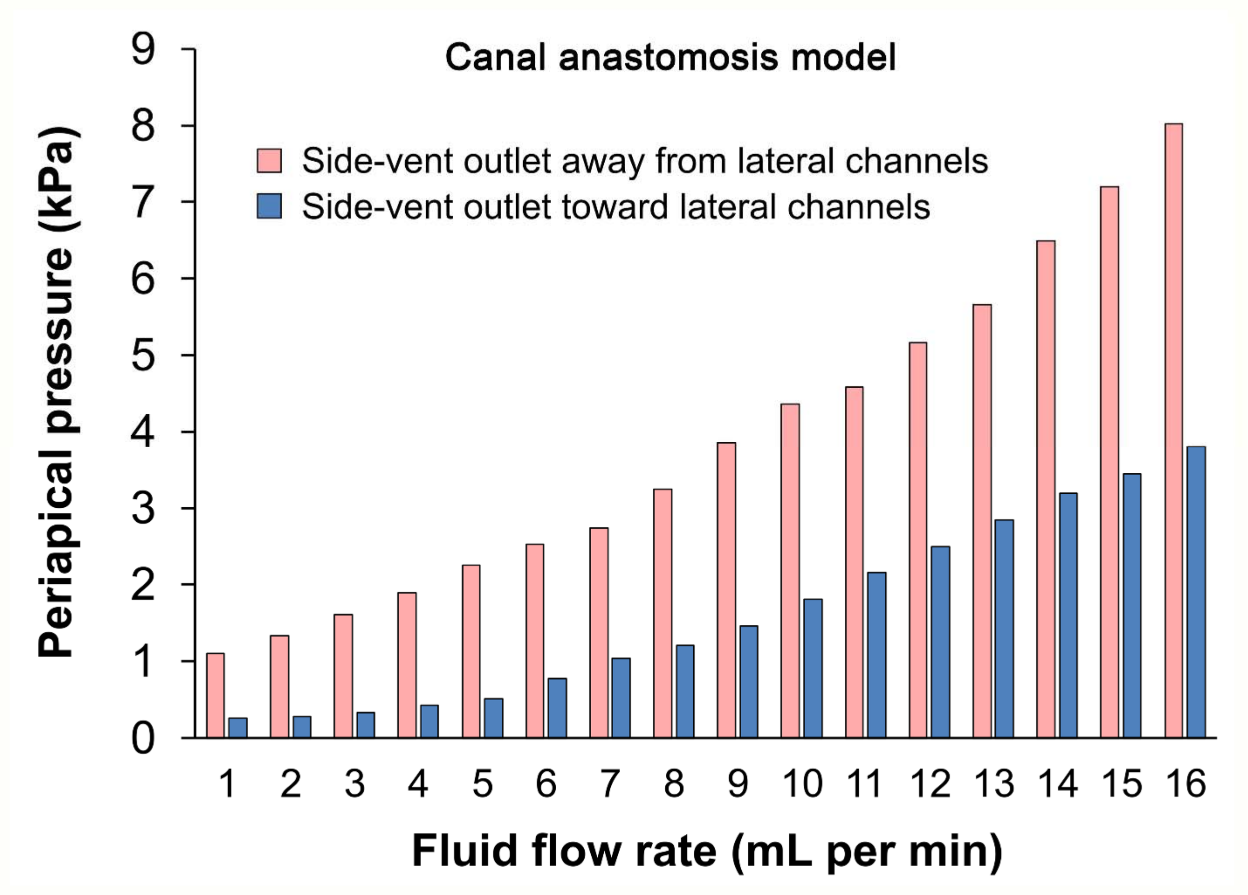

Figure S-3 The effect of side-vent outlet orientation on the development of periapical pressure in the canal anastomosis model. The 30-gauge side-venting needle to 1 mm short of the working length. In the pink series, the side-vent outlet was oriented 180° from the communicating channels. In the blue series, the side-vent outlet was facing the communicating channels directly.

Video Legends

Supplementary Video S-1a: Video corresponding to the microcomputed tomography image shown in Figure 1a of the main text (a single-rooted maxillary premolar containing two separate canals).

Supplementary Video S-1b: Video corresponding to the microcomputed tomography image shown in Figure 1b of the main text (a single-rooted maxillary premolar containing two separate canals connected by a lateral anastomosis in the mid-root).

Supplementary Video S-1c: Video corresponding to the microcomputed tomography image shown in Figure 1c of the main text (a single-rooted maxillary premolar containing two separate canals connected by a larger anastomosis in the mid-root).

Supplementary Video S-1d: Video corresponding to the microcomputed tomography image shown in Figure 1d of the main text (a single-rooted maxillary premolar containing Two separate canals connected by a sheet-like isthmus in the middle-third and a narrow anastomosis in the coronal-third of the root).
